# Engineered Faceted Cerium Oxide Nanoparticles for Therapeutic miRNA Delivery

**DOI:** 10.3390/nano12244389

**Published:** 2022-12-09

**Authors:** Yifei Fu, Elayaraja Kolanthai, Craig J. Neal, Udit Kumar, Carlos Zgheib, Kenneth W. Liechty, Sudipta Seal

**Affiliations:** 1 Advanced Materials Processing and Analysis Center, Dept. of Materials Science and Engineering, University of Central Florida, Orlando, FL 32826, USA; 2 Laboratory for Fetal and Regenerative Biology, Department of Surgery, College of Medicine, University of Arizona, Tucson, AZ 85721, USA; 3 Nanoscience Technology Center, Biionix Cluster, College of Medicine, University of Central Florida, Orlando, FL 32826, USA

**Keywords:** cerium oxide, hydrothermal method, miRNA, gene delivery, diabetic wound healing, nanomedicine

## Abstract

In general, wound healing is a highly ordered process, with distinct phases of inflammation, proliferation, and remodeling. However, among diabetic patients, the progression through these phases is often impeded by increased level of oxidative stress and persistent inflammation. Our previous studies demonstrated that cerium oxide nanoparticles (CNPs) conjugated with therapeutic microRNA146a (miR146a) could effectively enhance wound healing by targeting the NFκB pathway, reducing oxidative stress and inflammation. In the present study, we consider the potential effects of nanomaterial surface-faceting and morphology on the efficacy of miRNA delivery. Compared with octahedral-CNPs and cubic-CNPs, rod-CNPs exhibited higher loading capacity. In addition, in comparing the influence of particle morphology on wound healing efficacy, several markers for bioactivity were evaluated and ascribed to the combined effects of the gene delivery and reactive oxygen species (ROS) scavenging properties. In the cellular treatment study, rod-CNP-miR146a displayed the greatest miR146a delivery into cells. However, the reduction of IL-6 was only observed in the octahedral-CNP-miR146a, suggesting that the efficacy of the miRNA delivery is a result of the combination of various factors. Overall, our results give enlightenments into the relative delivery efficiency of the CNPs with different morphology enhancing miRNA delivery efficacy.

## 1. Introduction

Normal, healthy wound healing is a highly complex biological process sensitive to several cellular and molecular regulatory processes. Macroscopically, healing comprises four overlapping phases: coagulation, inflammation, migration-proliferation, and remodeling [1]. Impairment in the progression through any of these phases leads to compromised wound healing. Diabetes is marked by such unhealthy wound healing, with non-healing diabetic wounds being among the common complications [2].

Recent research has suggested that microRNAs (miR), a class of ~23 nucleotide-long single-stranded noncoding RNA molecules, are essential regulatory molecules involved in the synthesis of protein and regulation of gene expression in the different phases of impaired wound healing [3]. For example, our previous study has shown that the levels of miR146a, which shows a vital role in impaired wound healing process by modifying proinflammatory gene expression, was substantially reduced in diabetic wounds [4]. However, free miR are prone to degradation due to endonucleases in physiological fluids. Additionally, their negative charge hinders cellular uptake due to electrostatic repulsion by the negatively charged cell membrane [5]. Therefore, incorporation with a stable delivery vector is essential to successfully deliver active miR [6].

Recently, cerium oxide nanoparticles (CNPs) have acquired increased attention in biomedical research for their various enzyme-mimetic ROS scavenging activities (e.g., superoxide dismutase (SOD) [7], catalase (CAT) [8], oxidase [9], and phosphatase [10], etc.). Generally, two forms of cerium oxide can form: ceric oxide CeO_2_ (Ce^4+^) and cerous oxide Ce_2_O_3_ (Ce^3+^); with these states co-existing as a mixed valence composition in solid phase materials. However, at the nanoscale, surface oxygen vacancies are formed with greater densities, leading to a proportional increase in (near-)surface Ce^3+^ sites due to charge compensation. Furthermore, due to the low reduction potential (1.52 V) [11] of the Ce^3+^/^4+^ couple, Ce^3+^ and Ce^4+^ can easily undergo catalytical redox cycling; thereby, conferring unique regenerative antioxidant properties. Previous studies have demonstrated that the surface Ce^3+^/Ce^4+^ ratio of a given nanoparticle formulation is closely related to its specific enzymatic activities. Generally, CNPs with high Ce^3+^ exhibit superoxide dismutase-mimetic activity, while those with higher Ce^4+^ typically exhibit catalase activity [12]. Thus, CNPs can scavenge two of the more common reactive oxygen species (ROS) associated with excessive oxidative stress, known to occur in instances of diabetes and impaired diabetic wound healing. 

The bioactivity of CNPs is reported to be influenced by their size, shape [13], synthesis environment, and surface character [14]. For instance, the moderate aspect ratio of the rod-shaped nanoparticles is found to provide better orientation-dependent interaction with cell surfaces [15]. Additionally, smaller spherical particles are notified to exhibit a better cell membrane permeability compared to larger particles due to observed cell permeation via an energy-independent cell uptake pathway [16]. In the present study, we focused our attention on the impact of morphology. CNPs in various morphology have been synthesized by a one-step hydrothermal synthesis method. Three different shapes, nano-octahedra, nano-cube, and nanorod, have been conjugated with miR146a to explore if a connection existed between the nanoparticles morphology and the miR delivery performance in the perspective of designing better drug delivery strategy for impaired wound healing treatment.

## 2. Materials and Methods

### 2.1. Materials and Methods

Cerium chloride heptahydrate (Sigma-Aldrich, St. Louis, MO, USA), Ceric ammonium nitrate (Sigma-Aldrich), Sodium acetate (Sigma-Aldrich), Acetic acid (Sigma-Aldrich), Sodium phosphate (Sigma-Aldrich), Dimethyl sulfoxide (Sigma-Aldrich), 1,1′-Carbonyldiimidazole (Sigma-Aldrich), and Borate buffer Pack (Thermo Scientific, Waltham, MA, USA) were purchased from a commercial vendor, and it’s used without further purification. The cell culture flask, well plates and, dimethyl sulfoxide solvent (DMSO) were procured from Fisher Scientific. The custom design amine with and without fluoroprobe tagged miR146a was purchased from Integrated DNA Technologies IDT. Marine-derived macrophages (RAW 264.7 cells, ATCC^®^ TIB-71™), murine dermal fibroblasts (MDF), human umbilical vein endothelial cells (HUVEC), and their growth medium were purchased from ATTC (American Type Culture Collection, Manassas, VA, USA). The fetal bovine serum (FBS), endothelial cell supplement growth kit, 3-(4,5-dimethylthiazol-2-yl)-2,5-diphenyl tetrazolium bromide (MTT assay) powder and, live/dead assay were procured from Invitrogen. 

### 2.2. Different Shapes of CNPs Preparation

**Figure 1:**
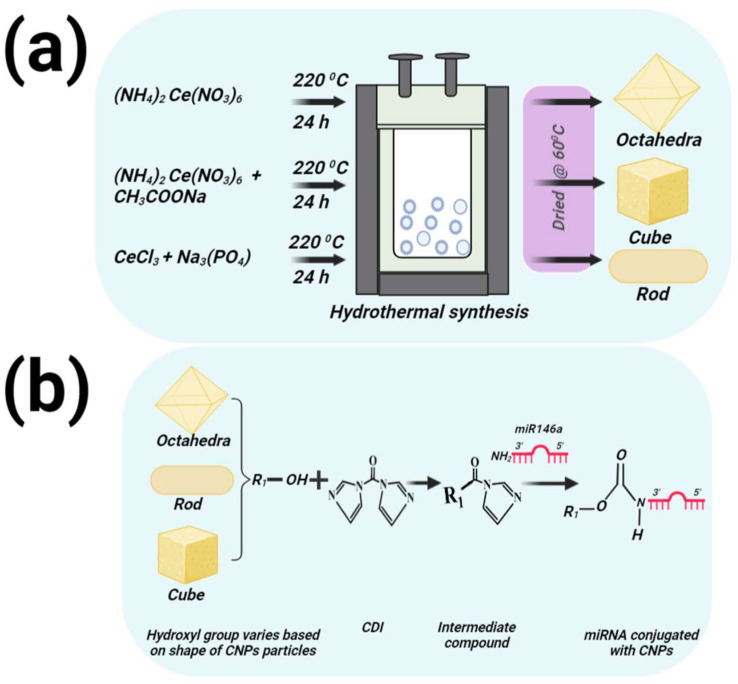
The schematic diagram represents the synthesis (**a**) of CNPs of unique morphologies and conjugation of miR (**b**) with CNPs of each formulation. CN_Oct_, CN_C_, and CN_R_ were synthesized under different reaction times and temperatures using a hydrothermal method.

Various shapes (Cube, rod, and octahedra) of CNPs were synthesized by a single wet chemistry hydrothermal method, while varying chemical precursor and/or thermal treatment time; a schematic diagram for the syntheses is shown in Figure 1a. The following steps were used to synthesize these nanoparticles. *Cube shape CNPs preparation*: 0.1 g of ceric ammonium nitrate ((NH_4_)_2_Ce(NO_3_)_6_) and 2.5 g of sodium acetate (CH_3_COONa) were dissolved in 17.5 mL of deionized water. A total of 2.5 mL of acetic acid was then added to the solution. The solution was stirred at room temperature for 1 h. Then, it was transferred to a Teflon-lined stainless-steel autoclave and subjected to hydrothermal treatment at 220 °C for 24 h. Afterward, the product was centrifuged, to allow the brown precipitate to separate, and subsequently washed three times with deionized water. Finally, the product was allowed to dry at 60 °C in a hot-air oven overnight. *Rod shape CNPs preparation*: Protocol was slightly adjusted from Ji [17]. study. A total of 0.6 mol of cerium (III) chloride (CeCl_3_) was dissolved in 15 mL in a beaker, and 0.02 mol of trisodium phosphate (Na_3_PO_4_) was dissolved in 5 mL of deionized water in another beaker. Then, 5 mL of Na_3_PO_4_ was added into 15 mL CeCl_3_ solution. The as-mixed precursor was vigorously mixed for 5 min before transferring the mixture into a Teflon-lined stainless-steel autoclave and subjected to hydrothermal treatment at 220 °C for 24 h. Afterward, the product was centrifuged, to allow the yellow precipitate to separate, and subsequently washed three times with deionized water. Finally, the product was allowed to dry at 60 °C in a hot-air oven overnight. 

*Octahedra shape of CNPs preparation*: A 15 mL solution of 0.6 M ceric ammonium nitrate was prepared. The solution was stirred in the open air for 15 min. Then, it was transferred to a Teflon-lined stainless-steel autoclave and subjected to hydrothermal treatment at 220 °C for 24 h. Afterward, the product was centrifuged, to allow the yellow precipitate to separate, and subsequently washed three times with deionized water. Finally, the product was allowed to dry at 60 °C in a hot-air oven overnight. The octahedron, cube and rod shape of the CNPs samples were herein after referred to as CN_Oct_ (CNP-octahedron), CN_C_ (CNP-cube) and CN_R_ (CNP-rod). 

### 2.3. MiR146a Conjugation with Different Shapes of CNPs

A schematic diagram for the miR146a conjugation with CNPs is shown in Figure 1b. A 30 μL aqueous solution of 1000 µg/mL octahedral, cubic, or rod-shaped CNPs were mixed with 270 µL of DMSO solution. A 30 µL solution of 500 mM CDI (1,1-carbonyldiimidazole) DMSO solution was then added. Next, the final solution was slightly agitated under room temperature for 1 h. A 50 µL solution of 200 µM miR146a aqueous solution was added into the activated CNPs solution. The resulting solution was gently shaken for 2–3 min by hand. Afterwards, a 3790 µL solution of 10 mM borate buffer, of pH of 8.5, was added followed by slight agitation under room temperature for 3 h. The resulting solution (2 mL) was transferred into the filter-insert part of each dialysis tube and was allowed to dialyze against RNAse/DNAse free water. It was then placed in an orbital shaker and continuously shaking at 150 RPM at room temperature. The water was replaced after 2 h and then left overnight. The miR conjugated CNPs sample was aliquoted and stored in a −20 °C until further use. The miR146a conjugated with CN_Oct_, CN_C_, and CN_R_ samples were named as CN_Oct_-miR146a, CN_C_-miR146a and CN_R_-miR146a. 

### 2.4. Characterization

X-ray diffraction (XRD) analysis was done on octahedra/cube/rod shape of CNPs by Empyrean Panalytical X-ray diffractometer using a CuKα (1.54 Å) radiation as a X-ray source. All X-ray diffraction patterns were analyzed used XPert Highscore software. X-ray Photoelectron Spectroscopy (XPS) was performed on before and after miR146a conjugation with three different shapes of CNPs. These samples were prepared for measurement via drop-casting particles from aqueous dispersion onto a cleaned gold substrate and drying under ambient conditions. XPS analysis was performed at room temperature using a Thermo Fisher spectrometer (ESCALAB-250Xi) under high vacuum (<1 × 10^−9^ mbar). A monochromatic Al-Kα source was implemented (15 kV, 20 mA). The beam spot size was 600 μm during measurement. Avantage (Thermo Scientific) software was used to analyze collected spectra; with the C1s peak referenced to 284.6 eV in fitted spectra. An Avantage *smart* background was applied to all analyzed spectra. Peaks were assigned to relevant material component chemical states by referencing published XPS data and analysis libraries (e.g., XPS simplified by Thermo Scientific). Cerium 3d spectra were fit by ten unique peaks (five spin-orbit doublets; Ce3d_5/2_, Ce3d_3/2_). TEM studies were carried out to study the morphologies before and after the conjugation of miR146a to CNPs. Holey carbon TEM grids (FEI) were used to prepare TEM samples. All samples were first dried on a grid at room temperature and then stored under a vacuum. ICP-MS sample preparation: 600 µL of the stock miR146a conjugated CNPs samples was digested for 48 h in 4 mL 35% nitric acid by heating at 80 °C in the conventional oven. Post digestion, the suspensions were diluted to a working suspension of a mass concentration of ~100 μg/L with ultrapure water, keeping nitric acid final concentration at 5% (for the sake of matching the background signal) in the solution. Further, all conjugated samples were characterized using UV-Vis spectrophotometry at room temperature in the wavelength range of 250 nm to 800 nm. 

#### 2.4.1. Catalase-Mimetic Activity (CAT)

To study the catalase mimetic activity of CNPs Invitrogen assay kit (Cat# A22188) (Amplex Red Hydrogen Peroxide/Peroxidase) was used. A FLUOstar Omega (BMG LABTECH) microplate reader was used read absorption values (excitation at 571 nm and emission at 585 nm). Following Beer’s law a standard curve for H_2_O_2_ was established and used to estimate the H_2_O_2_ concentration in each sample. A total of 50 μL of the sample, at a fixed common concentration for all samples, is preincubated with 50 μL of 2 μM H_2_O_2_ for 30 min in 96-well plates. Before the fluorescence readings were taken, 100 μL of working solution (100 μM Amplex Red reagent and 0.2 unit/mL HRP) was added and allowed to incubate at room temperature for 30 min. CAT activity is calculated by dividing the final H_2_O_2_ concentration in each sample by the initial H_2_O_2_ concentration and multiplying it with 100%.

#### 2.4.2. Superoxide Dismutase Mimetic Activity (SOD)

SOD activities for nanoparticles and nanoparticle conjugates of each synthesis method were measured using a home-built assay, reliant on a xanthine/hypoxanthine redox couple and WST-1 dye. In addition, the reduction of WST-1 was utilized to assess the kinetics of the reaction. The protocol was slightly adjusted from Peskin [18] study. Briefly, superoxide was generated by a 0.1 mM hypoxanthine/xanthine oxidase system. The generation rate of superoxide was controlled by adjusting the xanthine oxidase concentration, resulting in an absorbance change at 438 nm of approximately 0.025 units per minute. Each assay was conducted at room temperature for 10 min in a 96-well plate with a total volume of 240 μL containing 20 μL of the sample. Tris buffer 50 mM of pH 7.5 was used for all reactions. The SOD activity is calculated based on below equation:

SOD Activity = (slope of absorbance change of SOD blank-slope of absorbance change of specimen)/slope of absorbance change of SOD blank × 100%(1)

#### 2.4.3. Cytotoxicity Studies on miR146a Conjugated Nanoparticles

The biocompatibility of with and without miR146a conjugated different shape synthesized CNPs was examined using murine-derived macrophage cells (RAW 264.7). RAW cells were grown in T75 a flask at 37 °C in a CO_2_ incubator using DMEM (Dulbecco’s modified eagle medium). After 85% confluency, the cells were detached from the flask by the addition of 0.1% trypsin-EDTA solution. The cell layer was then scraped and isolated by centrifugation. Then, 3 × 10^4^ cells per well were added to a 96 well plate and incubated at 37 °C for 12 h in 5% CO_2_ air environment conditions to allow cell attachment to the plate. Then, media was removed and 0.4 ng/µL miR146a conjugated rod, Cube and octahedra CNPs were added to the wells and incubated for 24 and 48 h. After each time point, 20 µL of 5 mg/mL 3-(4,5-dimethylthiazol-2-yl)-2,5-diphenyltetrazolium bromide (MTT) solution was added to each well and incubated for 4 h in a CO_2_ incubator. The MTT solution was removed after 4h incubation, followed by adding 200 µL of DMSO to each well to dissolve the formazan crystals. It was continuously shaken for 30 min using an orbital shaker to make a homogeneous solution. Finally, the optical density of these solutions was measured at 570 nm in a BMG Labtech multi-plate reader. Cells grown cultured without nanoparticle treatment were considered as control. The experiment was conducted in triplicate for each condition. During the MTT assay, a live/dead assay was used to qualitatively prove the number of live and dead cells following the incubation period. A total of 0.4 µg/µL of free and CNPs conjugated miR treated cells were cultured and incubated for 48 h to perform the live/dead assay. Then, the medium was removed from the well and the cells stained using Molecular probes live/dead assay kit. Next, 2 mM of calcein and 4 mM ethidium homodimer solution were added to the cells, and the well plate was added and incubated at 37 °C for 30 min under CO_2_ atmospheric condition. After incubation, live and dead cell images were recorded using a Nikon fluorescence microscope. Passage ten cells were used for all experiments. 

#### 2.4.4. microRNA Delivery and Real-Time qPCR

HUVEC cells were used to qualitatively analyze the delivery efficacy of miR146a into cells. The VEGF-endothelial cell growth kit with vascular endothelial growth medium was used to culture the cells at 37 °C. The cells were detached at 80% confluency. The 1 × 10^4^ cells/well were seeded into 48 well plates. Following 4 h of incubation, 0.4 ng/µL of fluoroprobe-miR146a conjugated CNPs were added followed by an additional 12 h of incubation to allow miR-146a to transfect into cells. Subsequently, PBS was used to wash the cells twice. A Nikon fluoresce microscope was then used to image the fluoroprobe-miR delivered by the nanoparticles. To analyze gene expression, murine dermal fibroblasts (MDF) were used. A medium comprising Dulbecco’s modified eagle high-glucose (DMEM, Sigma-Aldrich, St. Louis, MO, USA) with 20% FBS (fetal bovine serum) + 1% antibiotic-antimycotic and maintained at 37 °C in a 5% CO_2_ humidified atmosphere was used to culture the MDF. The cells were seeded and cultured for 12 h for future experimentation. Afterwards, the cells were starved for FBS for 16 h followed by treatment with biocompatible 0.2 ng/μL pure and miR146a conjugated different shape of CNPs for 6 h. Total cellular RNA was isolated the cells using Qiazol (Qiagen, Hilden, Germany) to homogenize per the instructions of the manufacturer. To analyze IL-6 gene expression, the isolated RNA was turned into cDNA (Applied Biosystems RT kit, Waltham, MA, USA). A housekeeper gene, known GAPDH, was used for normalization. An additional set of RNA samples was diluted to 5 ng/µL through serial dilutions, and converted to miR146a and housekeeping RNA, U6, cDNA (Applied Biosystems RT kit). It was then amplified by reverse transcriptase amplification. A BioRAD CFX-9600 thermal cycler was used to perform a real-time quantitative polymerase chain reaction (RT-qPCR) for IL-6, GAPDH, miR146a, and U6. The PCR analysis was performed three times, with the averages used for normalization. To make multiple comparisons between various nanoparticle-treated samples, one-way ANOVA with Tukey’s test was used with statistical differences considered *p* < 0.05.

## 3. Results and Discussion

### 3.1. Structural and Compositional Analysis of Synthesized Nanoparticles

**Figure 2:**
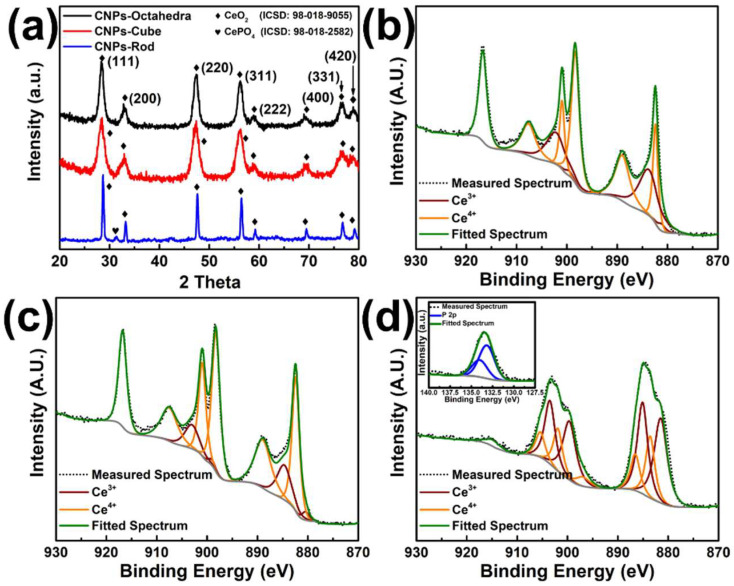
XRD and XPS of different shapes of CNPs were synthesized by the hydrothermal method. (**a**) shows the XRD patterns of CNPs-rod (bottom), CNPs-cube (middle), and CNPs-octahedra (top). The major peaks in the XRD for these nanoparticles correspond to the cubic structure of cerium oxide (ICSD: 98-018-9055) and the minor peak in the CNPs-rod XRD pattern corresponds to CePO_4_ (ICSD: 98-018-2582). This result suggested that the CeO_2_ structure has formed all conditions. Only, the CN_R_ synthesis condition produces the CeO_2_ along with traces of the CePO_4_ phases. (**b**–**d**) shows the deconvoluted Ce3d binding region in the XPS spectrum of CN_Oct_, CN_C_, and CN_R_, and (**d**)-insert shows the deconvoluted P2p region in CN_R_ samples. The deconvoluted peaks are clearly distinguished according to their oxidation states of Ce^3+^ (shown in red color) and Ce^4+^ (shown in orange color) in the CeO_2_ structure. Spectrum representatives for each sample are unique from each other: highlighting the distinct surface character of each morphology (e.g., faceting and vacancy site presentation). The percent of reduced cerium sites, relative to total cerium sites, was observed to increase with increasing material aspect ratio (26.8, 16.1, and 66.6% for CN_Oct_, CN_C_, and CN_R_, respectively).

X-ray diffraction studies were conducted to provide insight into particle crystallinity and structure within each formulation. As shown in Figure 2a, XRD patterns for formulations that produce CeO_2_ particles of CN_Oct_, CN_C_, and CN_R_ show eight significant peaks. These peaks can be indexed to the (111), (200), (220), (311), (222), (400), (331), and (420) planes of cubic CeO_2_ (ISCD 98-018-0955). Minor peaks belonging to CePO_4_ were found in the CeO_2_ rod. The lattice parameter was estimated for CNPs for all three particle formulations. As is shown in Figure 2a, the XRD patterns of CeO_2_-rod have been shifted to a higher theta value, signifying a lower lattice parameter (5.38 Å) compared to CeO_2_ octahedra (5.468 Å) and Cube (5.464 Å). Furthermore, if we observe the relative peak intensity in all three samples, (111) is the most pronounced, followed by (220) and (311), respectively. This observation is consistent with other studies and supported by the higher planar atomic density and lower surface energy of the (111) plane in the fluorite cubic system. Another qualitative observation can be made regarding the peak broadening effect. In CeO_2_, octahedra and cube samples have higher FWHM values for corresponding peaks than rod samples, suggesting a smaller crystallite volume. Therefore, a profile fitting analysis was performed on all the XRD pattern and Scherrer’s equation [19] was applied to determine the crystallite size (D) of CNPs, a brief section regarding the calculation is provided in Supplementary Materials page 3–4, with Table S1 and Figure S2. The calculated average D value was calculated and listed in Table 1, they are 7.8 ± 0.5, 5.5 ± 0.2, and 26.5 ± 1.5 nm for CN_Oct_, CN_C_, and CN_R_, respectively. The observed characters of all formulations collectively confirm the formation of CeO_2_ crystalline phases. From here, differences in material surface chemical compositions were considered via X-ray photoelectron spectroscopic analysis (XPS). In particular, the relative fraction of reduced state cerium sites within each formulation were considered as these sites are known to contribute strongly to the general surface catalytic character.

Analysis of Ce3d spectra for each sample (CN_Oct_, CN_C_, and CN_R_ in Figure 2b–d, respectively) reflect the mixed valency (Ce^3+^, Ce^4+^) demonstrated by cerium oxide nanomaterials. Spectra were deconvoluted and fit to peaks representative, specific cerium levels (*v*_0_, *u*_0_, *u*′, and *v*′ for Ce^3+^; *v*, *u*, *v*″, *u*″, *v*′″, and *u*′″ for Ce^4+^). From here, the fraction of reduced cerium sites, relative to the total measured cerium, was calculated via area under the curve integration and summation as

% Ce^3+^ = 100 * {∑∫(*v*_0_, *u*_0_, *u*, *v*″)}/{∑∫(*v*_0_, *u*_0_, *u*, *v*″) + ∑∫(*v*, *u*, *v*″, *u*″, *v*′′′, *u*′′′′′′)} (2)

The determined fractions of Ce^3+^ (Ce^4+^) were 26.8 (73.8), 16.1(83.9), and 66.6 (33.4) % for CN_Oct_, CN_C_, and CN_R_, respectively. It was observed that CN_R_ have substantially higher percentage of Ce^3+^ over the other morphology formulations. In addition, to Ce 3d, P 2p binding regions were measured with significant phosphorous signal identified in the CN_R_ spectra. The P 2p core level spectra (Figure 2d-insert) show two peaks located at 134.0 eV (P 2p_1/2_) and 133.1 eV (P 2p_3/2_) indicating the formation of trivalent phosphorus (PO_4_^3−^) in the sample. In the present study, the CN_R_ synthesis was chosen from a study by Ji [17], using CeCl_3_ as a cerium source and Na_3_PO_4_ as the mineralizer. It was noted in this study that formation of CePO_4_ impurity can be avoided by maintaining the Ce^3+^/PO_4_^3−^ (C/P) ratio above 5 at 1 × 10^−3^ M Na_3_PO_4_. However, in a study by Vinothkumar [20], it was observed that a CeO_2_-CePO_4_ composite phase formed at 10 C/P ratio. Considering that in the present study we use a much higher C/P ratio (120) in our CN_R_ synthesis and observe only a minor peak in XRD (Figure 2a), we assert that the high quantified Ce^3+^ surface content could be attributed to the formation of CePO_4_ regions among CeO_2_ regions. In the following section, particle morphology and average particle size are demonstrated through TEM. 

### 3.2. Size and Morphology Analysis of Synthesized Nanoparticles

**Figure 3:**
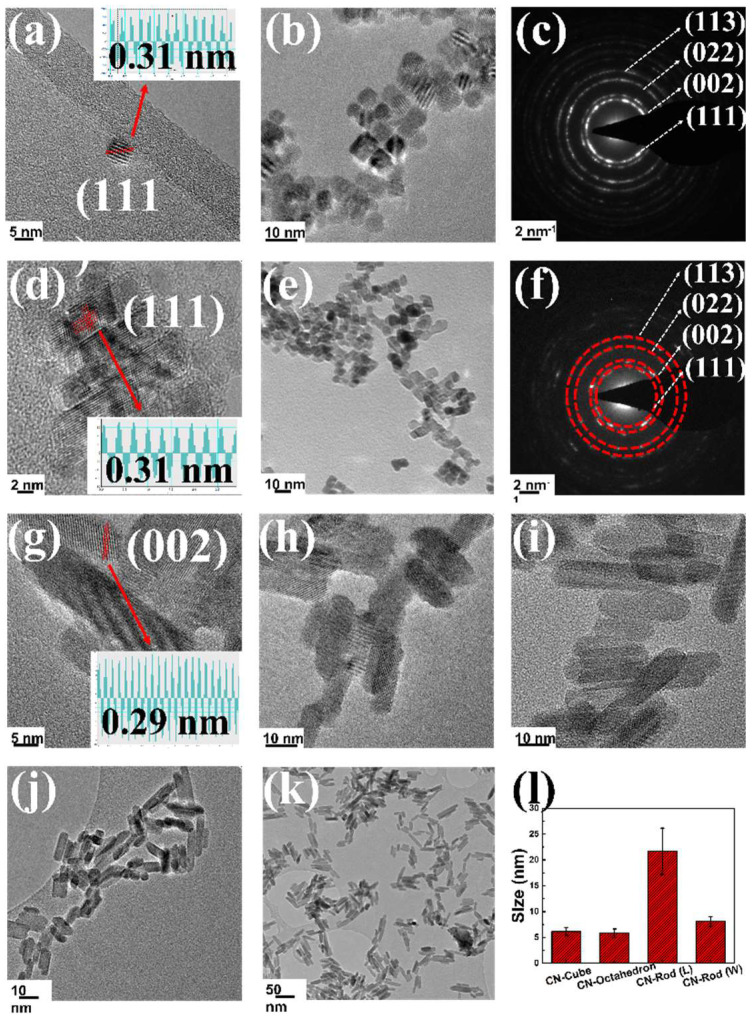
(**a**–**k**) Shows TEM analysis of CNPs of various morphologies. (**a**,**b**) show CNPs of cubic morphologies, with an average size of 6.13 ± 0.74 nm (estimated along the diagonals), and (**c**) shows the corresponding SAED pattern. (**d**,**e**) show octahedron-shaped CNPs with an average size of 5.8 ± 0.85 nm, and (**f**) shows a corresponding SAED pattern. (**g**–**k**) show rod-shaped CNPs with an average length of 21.6 ± 4.5 nm and a width of 8.1 ± 0.96 nm. (**l**) shows a bar graph of the size estimation of different morphologies of CNPs, Cubes, octahedrons, and rods. It can be observed in all three morphologies particle sizes are relatively uniform. In the case of rods, an average aspect ratio of 21:8 (length:width) has been demonstrated. The interplanar distance was estimated for one the rod sample (**g**), using Digital micrograph software, to be ~2.9 nm (likely related to the (002) plane). All other size estimation was performed using ImageJ software.

TEM imaging was done on different shaped CNPs to study particle size and morphology dispersion further. Figure 3a–l shows cubic, octahedrons, and rod particles with narrow size dispersion were obtained. Figure 3a,b are representative of the particles from CN_C_ formulation in relatively high-resolution images, showing cubic and slightly truncated cubic morphologies. Lower magnification images shown in Supplementary Materials Figure S1 give further understanding of overall size distribution. Selected area electron diffraction (SAED measurements in Figure 3c corroborate CeO_2_ structure demonstrated in XRD measurements). Similarly, Figure 3d displays high-resolution images of octahedron NPs. It should be noted that the rhombohedral appearance of the particles is a consequence of the octahedron and truncated octahedron morphologies projected into 2D in the presented and representative images. Low magnification images are also included in Figure 3e to relate size and morphology dispersion. A corresponding SAED (selected area electron diffraction pattern) is shown in Figure 3f. For rod particles, high-resolution images are shown in Figure 3g–i, while low magnification images are shown in Figure 3j,k. Unlike results from similar studies, we have obtained a nearly uniform size distribution. Figure 3l shows the size estimation of different particles produced from TEM images and ImageJ software (Version: Java 1.8.0_172 64-bit, Wayne Rasband and contributors, National Institutes of Health, Bethesda, MD, USA). Interplanar distance estimation was performed by focusing on single particles from each of the cube, octahedron and rod formulations. TEM micrographs suggested that the particles had a uniform size distribution, and the diameter of octahedra, cube, and rod shaped CNPs were 5.8 ± 0.85 nm, 6.13 ± 0.74 nm, and 21.6 ± 4.5 nm (length) 8.1 ± 0.96 (width), respectively. A slight difference was observed between size calculated by Scherrer’s equation from XRD and size measured from TEM image, a brief discussion is provided in Supplementary Information page 3–4, with Table S1 and Figure S2. Interplanar distance of exposed lattice fringe for cubic particles was estimated to be 0.31 nm [(111) planar system], for octahedron it is also 0.31 nm [(111) planar system], and for rod particles it was observed to be ~0.29 nm similar to the (002) planar system. Exposed facet would be the plane perpendicular to the exposed lattice fringe planar system. Therefore, for instance, the measured lattice spacing for CN_R_ particles is related to the exposed planes along the long axis of the rods. A technical, mechanistic discussion of particle shape evolution for each presented synthesis is included in Supplementary Materials for the interested reader Figure S5. To confirm the success of the conjugation process, bare and miR146a-conjugated particles from each formulation (CN_Oct_, CN_C_, CN_R_) were characterized by XPS, UV-vis, zeta potential, hydrodynamic size, and surface area measurements. 

### 3.3. Formulation Colloidal Stability and Influence of microRNA Conjugation

As shown in Figure S5 and Table 1, BET specific surface areas of the different shape CNPs are associated with the nanoparticles size and morphology. With the change of shape, the specific surface area changed accordingly. The particle size of CN_Oct_ and CN_C_ are similar (CN_Oct_: 5.8 ± 0.85 nm and CN_C_: 6.13 ± 0.74 nm), however the CN_C_ (116.97 ± 0.61 m^2^/g) have a higher surface area than that of the octahedra (98.65 ± 0.15 m^2^/g). Rod-shaped particles were larger than either other morphology (length 21.6 ± 4.5; width 8.1 ± 0.96) with an average specific surface area (63.28 ± 0.01 m^2^/g) lower than the other two shape formulations due to the significantly greater volume of individual particles. Based on the miR concentration and CNPs mass concentration obtained from ICP-MS study, the loading of miR146a per NPs was calculated and listed in Table 1. We observed that the CN_Oct_ particles showed the highest relative miR146a density followed by rods and cubes, respectively. Given that this trend does not correlate simply with the formulated particle specific surface area, we propose that the unique loading densities arise as a consequence of several surface properties. We observe that the loading density trend tracks with the particle Ce^3+^ fraction. This relation is reasonable considering the use of surface hydroxyl moieties in the conjugation process and the alteration of surface hydroxylation at the vacancy sites. Among the highest density crystal planes in the ceria lattice, {111} > {110} > {100} with respect to atomic density and overall stability. Therefore, an inverse trend in vacancy density (and vacancy energy) will be seen for these planes as lower density structures will more easily (at lower energy cost) accommodate vacancies. However, trends based on particle morphology can be confounded by effects from the chemical environment.

Octahedra particles from the CN_Oct_ formulation appear to be largely closed by {111} planes, suggesting the largest fraction of total Ce^3+^ sites lie on these planes. Some fraction of the total quantified reduced sites will also lie on facet edges, as well as other low-coordination sites (e.g., corners, steps), and the lower density ({110}, {100}) planes, however these sites are less pronounced in TEM images (Figure 3d,e). Interestingly, nanocubes are largely closed by {100} planes: suggesting a high tolerance for vacancies. However, measured reduced sites are significantly lower among CN_C_ particles compared to those of CN_Oct_. We assert that the lower number of reduced sites measured occurs as a consequence of surface binding of acetate species during synthesis. These small organic species confer greater stability to {100} planes, being lower density and allowing ordering of acetate species. Thereby, <100> growth is limited, leading to controlled particle size and morphology (as discussed in greater detail in Supplementary Figure S5) as well as decreased presence of cerium reduced sites due to acetate conjugation/stabilization. In the case of NC_R_, XPS results suggest that a large fraction of Ce^3+^ sites are related to cerium phosphate surface phase for CN_R_ particles, possibly confounding this correlation. 

Ce3d XPS and UV-Vis spectroscopy and dynamic light scattering (DLS) measurement were performed on CN_C_-miR146a, CN_Oct_-miR146a, and CN_R_-miR146a and the results are shown in Figure S5a–d and Table 1, respectively. No significant difference in Ce3d envelope were observed between bare CNPs and miR146a conjugated CNPs sample, indicating that Ce^3+^/Ce^4+^ ratio as well as the enzymatic activity of the conjugated sample were not impacted by the miR146a conjugation process. As shown in Figure S5d, a peak located at 300 nm is observable for all bare particle formulations, arising due to charge-transfer between Ce 4f and O 2p states. After conjugation, spectra were dominated by a peak located at ~260 nm caused by strong UV absorption from the heterocyclic rings associated with miR146a nucleotides. The hydrodynamic diameter and size distribution of the bare particles and conjugates were measured by DLS (dynamic light scattering). Both increased hydrodynamic size and change in zeta potential polarity, from positive to negative, indicated that particles from all three formulations were successfully modified by the negatively charged miR (Table 1). Zeta potentials for all measured samples were >|25| mV for all samples before and after conjugation, sparing CN_R_-miR146a (~−20 mV), suggesting strong colloidal stability overall. Additionally, the common polarity, and the charge suggests that electrostatic considerations for cell uptake behavior should be similar across formulations. Further, the spatial degrees of freedom allowed for the charge-determining miR species on the material conjugates should reduce the influence of varying loading efficiencies for different exposed facets. However, the relative curvature of the material surface for particle from each formulation are expected to contribute to uptake behavior.

### 3.4. Enzymatic Activity Assay (SOD and CAT)

Superoxide dismutase mimetic activity (SOD) was analyzed on pure and conjugated CNPs by WST-1 colorimetric assay of using multiplate reader. The superoxide radical anions (O_2_^●−^ ion) are generated by hypoxanthine-xanthine oxidase. This assay employs the hypoxanthine-xanthine oxidase system to generate O_2_^●−^ ion and oxidized WST-1 as a superoxide detection system.

Hypoxanthine + O_2_ → Uric acid + O_2_^●−^ (catalyzed by Xanthine Oxidase) (3)

The reduction rate of WST-1 by O_2_^●−^ is monitored spectrophotometrically at 438 nm. The reduction rate of WST-1 is inhibited when the SOD/SOD mimic containing sample is added due to the enzymatic dismutation of superoxide anion. The competition reaction between O_2_^●−^-SOD/SOD mimic and O_2_^●−^-WST-1 is measured as a decrease in the observed rate of the reduction of the WST-1, and the degree of decrease is proportional to the total SOD activity of the sample.

WST-1 + O_2_^●−^ → WST-1 Formazon + 2O_2_ (4)

2O_2_^●−^ + 2H^+^ → H_2_O_2_ + O_2_ (catalyzed by SOD or SOD mimic) (5)

The rate of the WST-1 reduction is measured in the absence of SOD/SOD mimic-containing sample (as SOD control) and with the presence of SOD/SOD mimic-containing sample (as test sample). 

Catalase-mimetic activity (CAT) was analyzed on pure and conjugated CNPs by the Amplex-Red assay of using multiplate reader. In the current study, CNPs with different morphology are incubated with 2 µM hydrogen peroxide in 96-well plates, followed by adding a solution of a mixture of the horseradish peroxidase and Amplex-Red into the 96-well plates. The fluorescent readings were taken to determine CAT activity by calculating the change of H_2_O_2_ concentration before and after CNPs/CNP-miR146a incubation.

**Figure 4:**
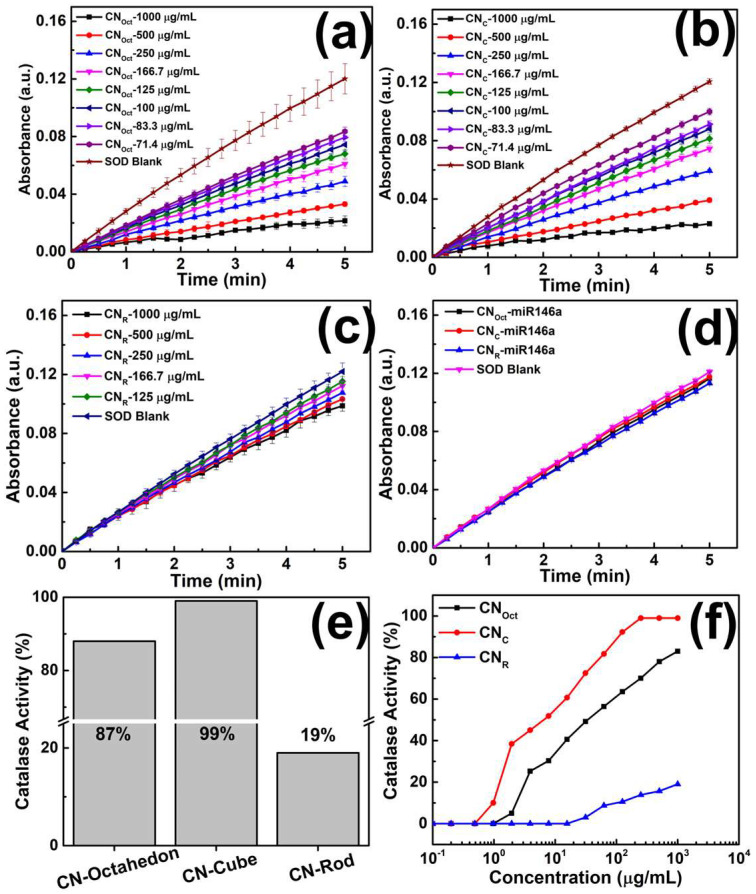
Superoxide dismutase (SOD) mimetic activity of CeO_2_ nanoparticles with different morphology at different concentration. Absorbance of sample, respectively, SOD control, CN_Oct_, CN_C_, CN_R_, (**a**–**c**), respectively, and miR146a conjugated all the samples of CNPs (**d**) at 438 nm is plotted as a function of assay reaction time. Linear fitting was used to measure the slope and calculate the SOD activity. CN_Oct_ exhibits the highest SOD activity. The miR146a conjugated CNPs showed very less SOD activity as compared to bare nanoparticles. It is due to below critical concentration of nanoparticles used for conjugation process. Additionally, the nanoparticles were losses during the purification steps using dialysis method. The catalase (CAT) assay was performed on bare and conjugated all shape of CNPs at room temperature using commercial catalase assay kit. Percentage of CAT activity calculated based on the residual H_2_O_2_ concentration after 20 min of incubation with CeO_2_ nanoparticles. Initial H_2_O_2_ concentration was set at 2 µM. (**e**) CAT activity of the CNPs stock solution, 1000 µg/mL. CeO_2_-octahedra and CeO_2_ cube exhibits substantial CAT activity, 87% and 99%, respectively, CeO_2_-rod exhibits mild CAT activity 19%. (**f**) CAT activity of CNPs at various concentration. Similarly, miR146a conjugated all shape of CNPs did not show the CAT activity due to the less concentration of nanoparticles present in the conjugated samples.

The calculated result of SOD and CAT activity are shown in Figure 4a–f: In SOD activity assay, the octahedra possesses the highest SOD activity 82.0%, followed by cube (77.2%) and rod (16.5%) at 1000 µg/mL concentration, in CAT activity assay, cube possesses the highest CAT activity 99%, followed by octahedra (87%) and rod (19%) at 1000 µg/mL (Figure 4a–d, Table 2). CAT (Figure 4e–f). (Note: Alternative comparison and discussion of the above SOD/CAT% assay results are carried out from a different perspective and provided in Supplementary Materials page 8–9, with Figure S6. The concept of “effective surface area” (S_ESA_), which associates with mass concentration (M), was introduced, and used to evaluate the enzymatic activity of the nanoparticles. Besides, a brief discussion on why M was chosen as the controlled variable in below discussion is also provided in the in Supplementary Materials page 10.)

As the SOD and CAT assay indicated that CNPs sample with concentration lower than 10 µg/mL would not show significant SOD or CAT activity, respectively, in present assay, none of the miR146a conjugates CNPs sample (1–6 µg/mL) show detectable SOD activity in the assay. Many of the theoretical and experimental study shows that the surface Ce^3+^/Ce^4+^ ratio in CNPs is highly related to its enzymatic activity as it may act as a catalytically active site [21,22]. In general, CNPs with high Ce^3+^ on the surface favor SOD mimic activity while CNPs with high Ce^4+^ on the surface favor CAT mimic activity [22]. Interestingly, in the present study, although the SOD and CAT activity of octahedral and cubical shape CNPs has a positive correlation relationship between surface Ce^3+^ and SOD activity (SOD: Oct > Cube, Ce^3+^: Oct > Cube) as well as surface Ce^4+^ and CAT activity (SOD: Cube > Oct, Ce^4+^: Cube > Oct), CN_R_ shows the lowest enzymatic activity in either SOD or CAT activity assay even though it possesses the highest Ce^3+^ among all CNPs sample in the present study. From the XPS discussion in the previous content, P 2p core level spectra implies the formation of trivalent phosphorus (PO_4_^3−^) in the sample since low concentration of Na_3_PO_4_ was introduced as a mineralizer and shape control additive in the synthesis and therefore the calculated high Ce^3+^ on rod nanoparticles surface could be a combined result of Ce^3+^ from both CeO_2_ regions and CePO_4_ regions, therefore, it is highly possible that the Ce^3+^ of rod do not follow the trend followed by octahedron and cube. Besides, many previous studies has reported that the formation of strong coordination complexes between Ce^3+^ and PO_4_^3−^ could diminishes the enzymatic activity [8,23,24]. Moreover, the PO_4_^3−^ groups coordinates with Ce^3+^ sites to create the complex CePO_4_/CeO_2_ crystal structure which reduces the enzymatic activity [23,24].

Interestingly, regarding the shape-dependent or exposed facet-dependent enzymatic activity study, many experimental studies hold different opinion and conclusion. Study from Li reported that octahedral and rod shaped CNPs have no SOD mimetic activity [25]; another finding has shown that in various shape of CNPs (polyhedra, cube and rod), all having approximately 70% Ce^3+^, SOD mimetic activity or CAT mimetic activity will be enhanced in CNPs exposing {100} or {111}, respectively [26]; a comparable study of Yang has indicated that with same level of Ce^3+^/Ce^4+^ ratio and oxygen vacancies on the surface of cubical and rod shaped CNPs, rod CNPs exposing {110} facets has was four times higher SOD mimetic activity than cubical CNPs with exposed {100} facet and cubical CNPs has 23 times higher peroxidase mimetic activity than rod shaped CNPs [27]. Therefore, since the Ce^3+^/Ce^4+^ of CNPs in present study is different between octahedral, cubical and rod shaped CNPs limited by need of further modification of the synthesis condition, it is reasonable to speculate that the both the exposed facet and the Ce^3+^/Ce^4+^ ratio plays an importance role on contributing the redox enzyme mimetic activity difference between various shape CNPs.

### 3.5. Biological Studies

#### 3.5.1. Biocompatibility Studies

**Figure 5:**
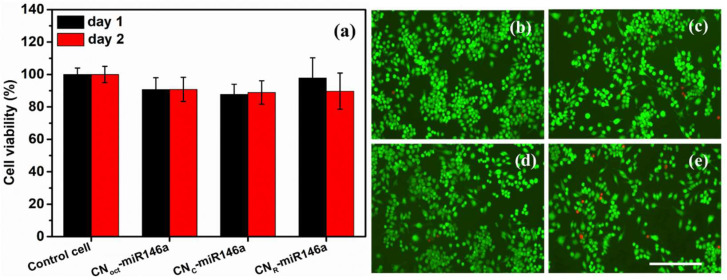
Cell viability and live/dead assay results of pure and miR146a conjugated octahedra, Cube, and rod CNPs samples. (**a**) shows the percentage of cell viability after 0.4 ng/µL of miR146a conjugated different shapes of CNPs-treated cells. Non-treated cells were considered as the control. In addition, the merged image of live and dead cells for control cells (nanoparticles non-treated cells), and 0.4 ng/µL of miR146a conjugated different shapes of CNPs were shown in (**b**–**e**). The scale bar is 100 µM.

The cytotoxicity of pure and miR146a conjugated different shapes of nanoparticles was analyzed using murine-derived macrophage cells (RAW 264.7). The toxicity of 0.4 ng/µL of miR146a conjugated CNPs treated cells was quantified using MTT assay and, as shown in Figure 5a. The toxicity result showed that miR146a conjugated with different shapes of CNPs did not show noticeable changes in cell viability over 48 h compared to non-treated cells. The results suggested that there is no substantial change in the cytotoxicity of miR146a conjugated CNPs. Further, live, and dead cell images were obtained for nanoparticles-treated and non-treated cells using the molecular probe live/dead assay kit. No significant change in the live cells percentage compared to control cells was observed (Figure 5b) and these results are consistent with the MTT assay results. This result is further proof of the cytocompatibility of the formulation.

**Figure 6:**
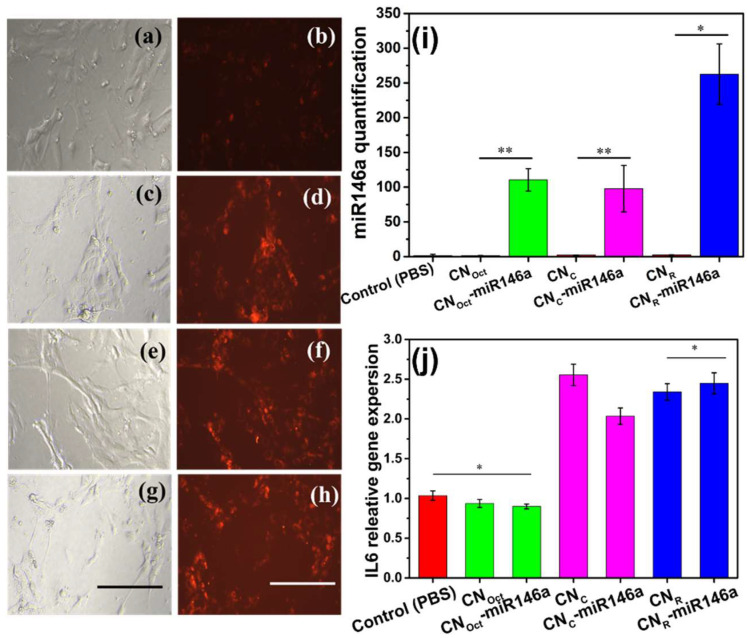
The bright field/fluorescence images, miR146a quantification and, IL6 relative gene expression were analyzed on miR146a conjugated in different shapes of CNPs. Control cells and 0.4 ng/µL of fluoroprobe tagged miR146a conjugated octahedra, rod, and cube CNPs treated cells bright filed and fluorescence image were shown in the image (**a**–**h**). The scale bar is 100 µm. (**i**,**j**) miR-146 and IL-6 gene expression following treatment of MDF with pure and miR-146a conjugated different shapes of CNPs. miR-146a levels were significantly higher in miR146a different shape CNPs than in control (PBS) treated sample (**i**). Octahedra CNPs and miR146a-octahedra CNP treated cells considerably reduced IL-6 gene expression compared to control (**j**). Bars indicate statistical significance with *p* < 0.0001 ** and *p* < 0.05 *.

Further, we have analyzed the delivery efficiency of miR146a into the cells using the qualitative fluorescence image technique. Fix concentration of fluoroprobe tagged miR146a conjugated samples were treated and incubated for 12 h. Figure 6c,e,g shows that the bright field and fluorescence microscope image clearly demonstrate successful delivery of miR146a into cells with the help of nanoparticles. Compared to control cells, nanoparticles transport more miR into cells (Figure 6b,d,f,h). 

#### 3.5.2. microRNA Delivery and Real-Time qPCR

The expression of miR146a and IL-6 were analyzed using MDF cells treated with pure and miR146a conjugated with various CNPs shapes. The MDF cells were treated with 0.2 ng/µL of biocompatible pure and miR146a conjugated with different shaped CNPs for 6 h. Afterward, the cells were processed and quantified. The relative miR146a expression is shown in Figure 6i. Sodium phosphate buffer (PBS) was considered as a control. Based on Figure 6i, the CN_R_ conjugated miR146a sample showed the greatest relative miR146a expression, indicating that the miR146a cellular delivery is affected by the shape of the CNPs it is loaded upon. The most plausible reason for the miR expression level difference between octahedron, cube, and rod shaped CNPs could be attributed to the nanoparticles shape-dependent cell internalization. It has been indicated in the previous study that nanoparticles uptake by cells through endocytosis is usually determined by complicated interplay of NPs physicochemical properties. 

It is well accepted that the primary mechanism for NPs cell penetration involves a series of endocytosis processes, which can significantly affect the targeted delivery of NPs and the carried therapeutic miRNA [28,29]. Multiple strategies have been developed to identify the type of endocytosis involved in NPs cell penetration. For example, it could be determined by performing a series of experiments either using multiple inhibitor molecules that prevent endocytosis [28] or by pretreating cells with different agents which deplete various endocytosis mediating plasma membrane species. In our previous study [30], we used the second mentioned strategy to demonstrate that the cellular uptake of CNPs is an energy-dependent process, which was mediated via clathrin-dependent endocytosis from lipid rafts.

As reported by Aberg reported that nanoparticle uptake comprises two steps. Firstly, the adherence of nanoparticles on the surface, and, secondly, the internalization of nanoparticles via energy-dependent endocytosis [31]. In the previous study, researchers have proven that relatively larger surface area of the elongated rod shape nanoparticle could facilitate the multivalent interaction between the nanoparticles and cell surface. Hence, it leads to a higher cell adhesion and internalization efficiency [32]. On the other hand, particles with sharp edges or high curvature, such as octahedral and cubical shape nanoparticles, can evade endocytosis leading to only a moderate uptake by the cells [33]. After the particles are physically attached to the cell membrane, endocytosis is started by the cellular membrane wrapping on the nanoparticles surface which occurred as a result of the competition between the stretching energy and the bending energy of the cell membrane [34]. As the octahedral and cubical shape particles contains more sharp edges compared to the rod, they might have to overcome a higher membrane bending energy barrier. However, when it comes to rod, a low membrane bending energy is required when the tip of rod-shaped NPs makes the initial contact [35], the launching mode. Besides, when the rod encounters the membrane via the submarine mode (initial contact made by elongated side), the membrane interaction again could be favored by the relatively flat and large surface area [35]. On the other hand, the bending energy of the membrane is to be also larger when wrapping a small particles, octahedron 5 nm and cube 6 nm, which radius is below critical size resulting in a low uptake ratio compared to rod which has a moderated size, 20 nm [34]. Further, the difference in nanoparticles cell internalization can also be interpreted based on their net surface charge values. It has been widely studied in the literature that both membrane disruption and penetration increase with increasing charge density [36]. The zeta potential values show that all the CNP-miR146a nanoparticle conjugates are negatively-charged at the surface, |CN_C_ (−33.0 eV)| > |CN_Oct_ (−25.5 eV)| > |CN_R_ (−20.0 eV)|. Another possible contributing factor to the observed trends in miR expression (CN_R_ produces a more substantial result than the other formulations) could be the effects electrostatic repulsion between particles and the negatively-charged plasma membrane. 

To analyze the proinflammatory gene expression, IL-6, RNA was isolated from the treated cells followed by cDNA synthesis (Figure 6j). IL-6 expression was shown to increase after a 6 h treatment of pure, rod, and cube shaped CNP-miR146a samples. However, IL-6 gene expression decreased in the CN_Oct_-miR146a sample when compared to the control, showing that bare CNPs can also reduce the expression of proinflammatory cytokine with a further reduction of IL-6 gene expression observed for the conjugate CNP-miR146a sample. There were no significant changes in the relative IL-6 gene expression in the CN_R_-miR146a and CN_R_-miR146a samples. The obtained results suggest that modulation in IL-6 expression, among the tested formulations, may result from the combined effects of cellular membrane penetration efficiency, ROS scavenging, and effects from miR-loading. CN_Oct_ and CN_C_ both demonstrated substantial ROS scavenging (both catalase and SOD activity), though produced negligible increases in miR expression. Based on the significant differences in IL-6 modulation, it is suggested that particles from the CN_Oct_ formulation have a greater cell permeation than CN_C_. This trend in small, octahedral (approximately spherical) being more efficient in permeating the cell membrane, over cube-shaped particles, is supported in published literature. However, it should also be noted that that miR-loading efficiency for CN_C_ is 15% less than that of CN_Oct_. In contrast, CN_R_ particles potentiated IL-6 expression, demonstrated negligible ROS scavenging, and produced substantial miR146a expression. We propose that while the observed increase in miR suggests the highest cell permeation and delivery efficiency, the high miR delivered per particle for this formulation may induce some toxic effect. Further, the negligible measured ROS scavenging activity of these particles suggests limited conferred cytoprotection by enzyme-mimetic ROS amelioration.

## 4. Conclusions

In the current study, we sought to identify variables that may affect the miR Delivery efficacy in using CNPs as the carrier in in vitro wound healing model. Single-step hydrothermal synthesis with various shape control agents was used to produce octahedral, cubic, and rod-shaped CNPs with uniform size distribution. Correspondingly, characterization of the physical properties of CNPs such as Ce^3+^/Ce^4+^ ratio, exposed facet, size, and zeta potential were performed, and the result demonstrated that rod shape CNPs with the highest Ce^3+^ percentage (66.6%) and surface charged possesses the best miR loading capacity. In addition, the biocompatibility test has shown that all shapes of CeO_2_-miR146a conjugate formulations were biocompatible: producing negligible cytotoxicity and effectively transferring the surface-conjugated exogenous miR146a as evidenced via PCR results. Furthermore, the observed difference in gene expression of various-shaped CNP-miR16a underscores the importance of morphologies and surface faceting of CNPs in therapeutic efficacy. Overall, our study provides an experimental basis for the application of shaped CNPs in effective diabetic wound healing regulation.

## Figures and Tables

**Table 1:** The physicochemical properties (particle size, zeta potential, and specific surface area) of pure and conjugated samples and the loading number of miR146a calculated based miR concentration measured by Quant_iT_microRNA_Assay and CNPs concentration measured by ICP-MS.

Sample	Particles Size (nm) from TEM	Crystalline Size (nm) from Scherrer’s Equation	Hydrodynamic Diameter (nm)	Zeta Potential (mV)	Specific Surface Area (m^2^/g)	Loaded miR146a (Strand/Particles)
CN_Oct_	5.8 ± 0.85	7.8 ± 0.5	221.3 ± 52.0	+30.2 ± 1.1	98.7 ± 0.15	(-)
CN_C_	6.13 ± 0.74	5.5 ± 0.2	237.6 ± 32.2	+26.9 ± 1.0	117.0 ± 0.61	(-)
CN_R_	21.6 ± 4.5	26.5 ± 1.5	307.7 ± 76.8	+32.5 ± 1.4	63.3 ± 0.01	(-)
CN_Oct_-miR146a	(-)	(-)	245.5 ± 3.3	−25.5 ± 8.8	(-)	~100
CN_C_-miR146a	(-)	(-)	447.2 ± 28.0	−33.0 ± 3.6	(-)	~85
CN_R_-miR146a	(-)	(-)	553.7 ± 23.5	−20.0 ± 3.1	(-)	~1200

**Table 2:** The percentage of SOD activity of various concentration of pure different shape nanoparticles and miR conjugated different shape of nanoparticles.

Concentration of Nanoparticles Used in the Assay	% SOD Activity		
Bare NPs	CN_Oct_	CN_C_	CN_R_
71.4 µg/mL	31.6	17.4	(-)
83.3 µg/mL	34.8	25.4	(-)
100 µg/mL	39.3	27.4	(-)
125 µg/mL	43.9	33.0	3.1
166.7 µg/mL	50.6	38.7	7.2
250 µg/mL	59.5	50.9	11.4
500 µg/mL	73.0	67.5	14.1
1000 µg/mL	82.0	77.2	16.5
**Conjugated** **NPs**	**CN_Oct_-miR146a**	**CN_C_-miR146a**	**CN_R_-miR146a**
1–6 µg/mL *	3	3	1

* CNPs concentration in conjugated samples quantified by ICP-MS technique.

## Data Availability

Not applicable.

## Supplementary Materials

The following supporting information can be downloaded at: https://www.mdpi.com/article/10.3390/nano12244389/s1, Figure S1: TEM image of octahedral/cubic shaped CNPs under different magnification; Table S1: The crystallite size (D) calculated by Scherrer’s equation; Figure S2: Marked peaks used in crystallite size calculation; Figure S3: Schematic diagram for the shape-controlled synthesis of CNPs nano-octahedron, nano-cube, and nano-rod; Figure S4: 27-point nitrogen adsorption isotherm of the octahedral/cubic/rod-shaped CNPs; Figure S5: UV-Vis and Ce3d XPS spectrum of miR conjugated CNPs; Figure S6: SOD and CAT% was plotted versus SCe^3+^. References [17,19,20,25,26,27,37,38,39,40,41,42,43,44,45,46,47] are cited in Supplementary Materials.
