# Supplementary material for: Engineered Faceted Cerium Oxide Nanoparticles for Therapeutic miRNA Delivery
Source: Nanomaterials (Basel). 2022 Dec 9;12(24):4389. doi: 10.3390/nano12244389 (PMC9784897; doi:10.3390/nano12244389)
Supplement: Supplementary file 1 [file nanomaterials-12-04389-s001.zip › nanomaterials-2073235-supplementary.pdf]

Supporting Information For:

Engineered Faceted Cerium Oxide Nanoparticles for Therapeutic miRNA Delivery

Yifei Fu[1], Elayaraja Kolanthai[1], Craig J. Neal[1], Udit Kumar[1], Carlos Zgheib[2], Kenneth W. Liechty[2], and Sudipta Seal[1,3]*

[1]*Advanced Materials Processing and Analysis Center, Dept. of Materials Science and Engineering, University of Central Florida, Orlando, FL, USA*

[2]*Laboratory for Fetal and Regenerative Biology, Department of Surgery, College of Medicine, University of Arizona, Tucson, AZ*

[3]*College of Medicine, Nanoscience Technology Center, Biionix Cluster, University of Central Florida, Orlando, FL, USA*

*Correspondence: Sudipta.Seal@ucf.edu; Tel.: +1-407-823-5277

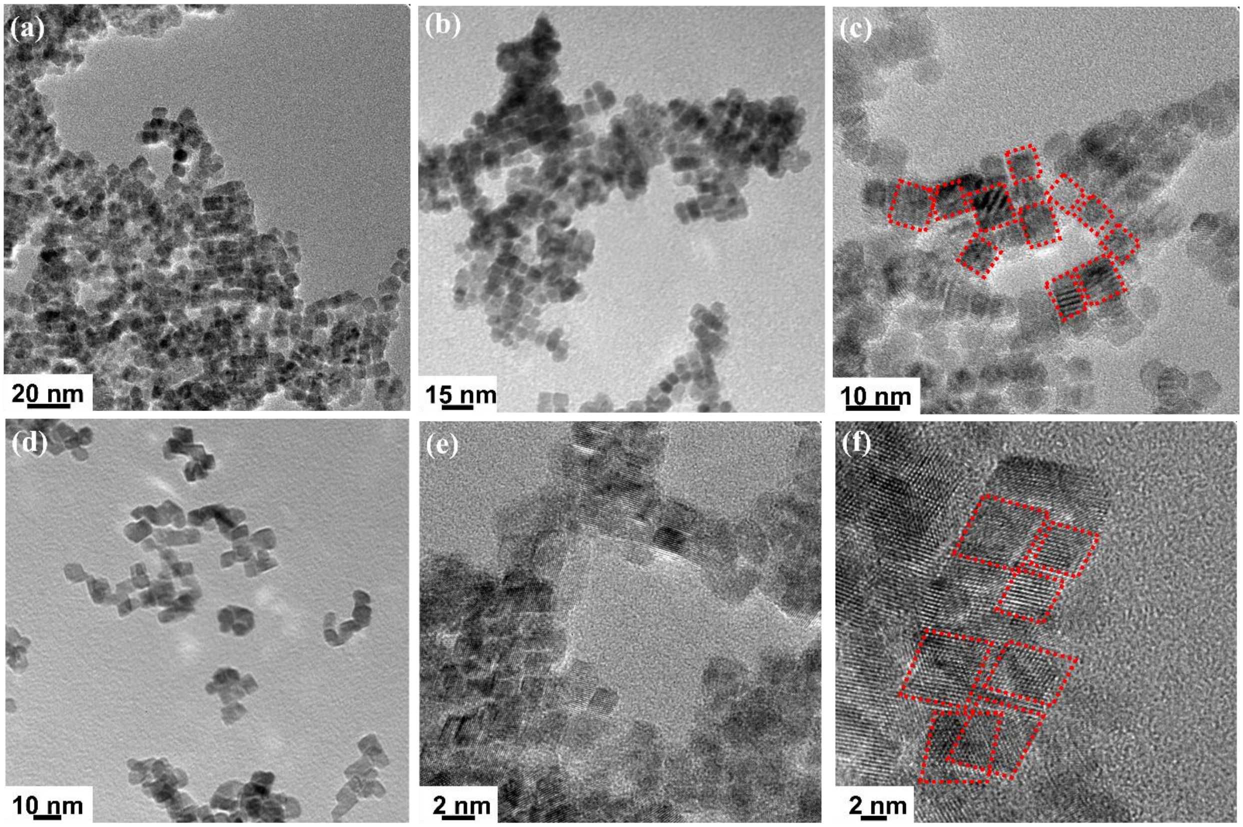

Figure S1. (**a-c**) TEM image of cubic shape cerium oxide nanoparticles under different magnification. Clear cubic morphology was observed for the majority of the nanoparticles with an average size of 6.13 ± 0.74 nm. (**d-f**) TEM image of octahedral shape cerium oxide nanoparticles under different magnification. Clear octahedral morphology was observed for the majority of the nanoparticles with an average size of 5.8 ± 0.85 nm.

.

Table S1. The crystallite size (D) calculated by Scherrer's equation.

Name of Peak	Miller Index (hkl)	B_{hkl} (°) Full-Width-Half-Maximum	2θ (°) 2 Theta	Calculated Crystallite Size (nm)
CN_{Oct}-1	(111)	1.003	28.385	8.2
CN_{Oct}-2	(200)	1.180	32.979	7.0
CN_{Oct}-3	(220)	1.102	47.290	7.9
CN_{Oct}-4	(311)	1.140	56.123	7.9
CN_{C}-1	(111)	1.508	28.344	5.4
CN_{C}-2	(200)	1.559	32.792	5.3
CN_{C}-3	(220)	1.564	47.174	5.5
CN_{C}-4	(311)	1.582	56.081	5.7
CN_{R}-1	(111)	0.310	28.676	26.5
CN_{R}-2	(200)	0.337	33.209	24.6
CN_{R}-3	(220)	0.308	47.582	28.2
CN_{R}-4	(311)	0.337	56.418	26.8

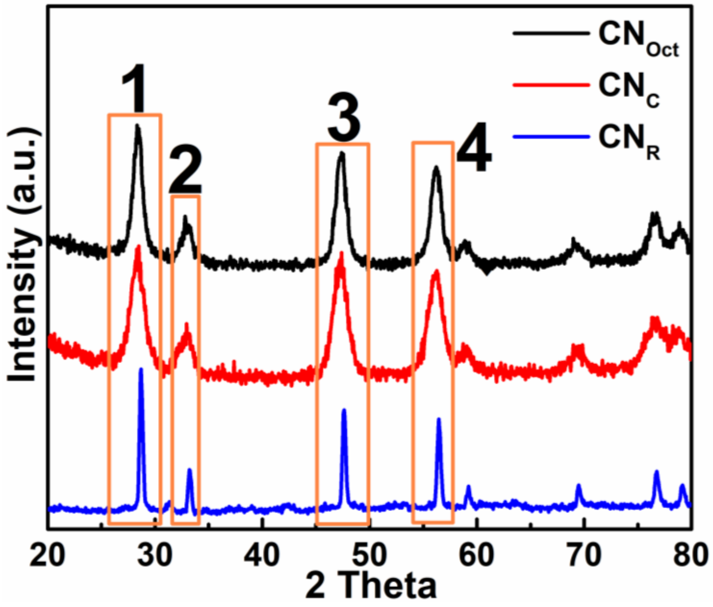

Figure S2. Marked peaks used in crystallite size calculation.

Crystallite size calculation using Scherrer's equation

In order to apply Scherrer's equation[1]

$$D_{hkl}=K^*\lambda/(B_{hkl}{}^*\cos\theta),$$

to calculate the crystallite size (D), a profile fitting analysis was performed to the XRD pattern of CN_{Oct}, CN_C, and CN_R respectively to measure the B_{hkl}, full-width-half-maximum of the diffraction peak, and θ, Bragg angle of the diffraction peak, using XRD analysis software HighScore Plus (v.4.5). λ is determined by the type of anode material, in present study, 1.540598Å (Cu). K, shape factor, was set at 0.9, as it has been comprehensively discussed in Langford and Wilson's previous study [2] that K does not display a wide variation with crystallite shape and a value of 0.9 could be taken as a first approximation value. As the instrumental broadening increases and peak intensity decreases at higher angle[3], only the first four peaks from the XRD pattern were selected to calculate the D.

As shown in Table S1 and figure S2, the calculated average crystallite size is 7.8, 5.5, and 26.5 nm for CN_{Oct}, CN_C, and CN_R respectively. Compared with the particle size measured from transmission electron microscopy (TEM) image, 5.8, 6.13, and 21.6, the crystallite size calculated by Scherrer's equation did not precisely coincide with the particle size measured from TEM. Considering that measurement, computational, and observational error from XRD profile fitting analysis and TEM image analysis may have an impact on the obtained result and, however, these results are in the same order of magnitude, we consider that the observed slight deviation is within acceptable error range.

The mechanism of the shape-controlled synthesis of CNPs

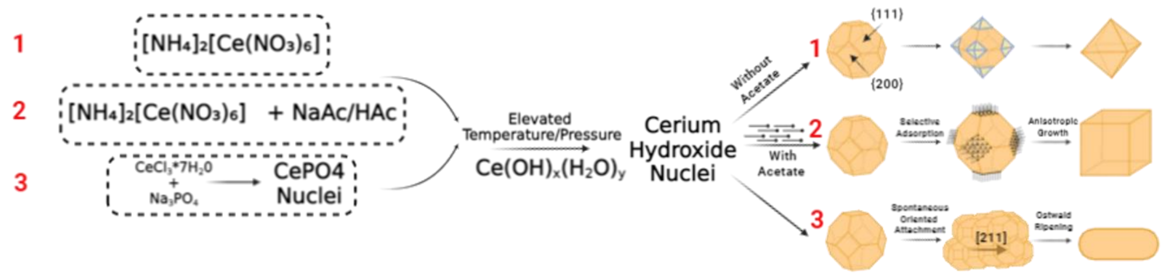

Figure S3. Schematic diagram for the shape-controlled synthesis of CNPs nano-octahedron, nano-cube and nano-rod.

Shape control growth mechanism: Previously, many studies have been conducted to prepare CeO_2 nanoparticles of varied morphologies in alkaline environment by using $Ce(NO_3)_3$*$6H_2O$ as a cerium source and NaOH or NH_4OH as the precipitating agent. In the present study, ceric ammonium nitrate (CAN) was the only chemical agent utilized in nano-octahedra synthesis. Both CAN and sodium acetate/acetic acid were used to form the CNPs-cube with acetate ion serving as a capping agent. Under hydrothermal treatment, initial transformation from aqueous CAN precursor to CNPs-nano-octahedra was initiated by the formation of dimer complex by two $Ce(NO_3)_6^{2-}$ octahedrons[4]. Due to the increased concentration of dimer during the synthesis, dimers assemble into larger clusters providing a nucleation site and the water molecules coordinated to the Lewis acid Ce^{4+} ion will tend to deprotonate, leaving hydroxide ion ligands coordinating with Ce^{4+} metal centers as $Ce(OH)^{3+}$ species interconnected by OH bridges. Generally, when Ce (III) salt is used, it requires the oxidation of Ce^{3+} to Ce^{4+} in solution. Correspondingly, the formation of nuclei of the hydration and hydrolysis of Ce4+ ions is limited by Ce^{3+}/Ce^{4+}oxidation, rate and nuclei formation. However, when using Ce (IV) salt in the synthesis, oxidation is no longer necessary, the precipitation from the Ce (IV) and the nucleation step could be faster which eventually leads to a finer and faster nucleation of the hydrate $Ce(OH)_x(H_2O)_y$ compared to Ce (III) salt synthesis. These small and extensively hydroxylated oligomers undergo dehydration at elevated temperature and eventually form cerium oxide nano-octahedra. This morphology exposes low surface energy, high density (111) planes, with this facet being the most stable among the three lowest index planes for Fluorite structured CNPs ((111)>(110)>(100)). In the CNP-nanocube synthesis, sodium acetate/acetic acid were added as a surface modifying agents which preferentially bind to the [5] {100} family of planes: reducing the growth rate of these facets and leading to a cubic morphology. As for CNPs-rod synthesis, studies from Ji[6] and Vinothkumar[7] each suggested that the formation of $CePO_4$ precipitates serve as nucleation sites for the subsequent growth of $Ce(OH)_3$ generated through hydrolysis of precursor $CeCl_3$. Eventually, the CNPs-nanorods are obtained from the self-assembly of cerium oxide nanoparticles along [211] direction followed by Ostwald ripening.

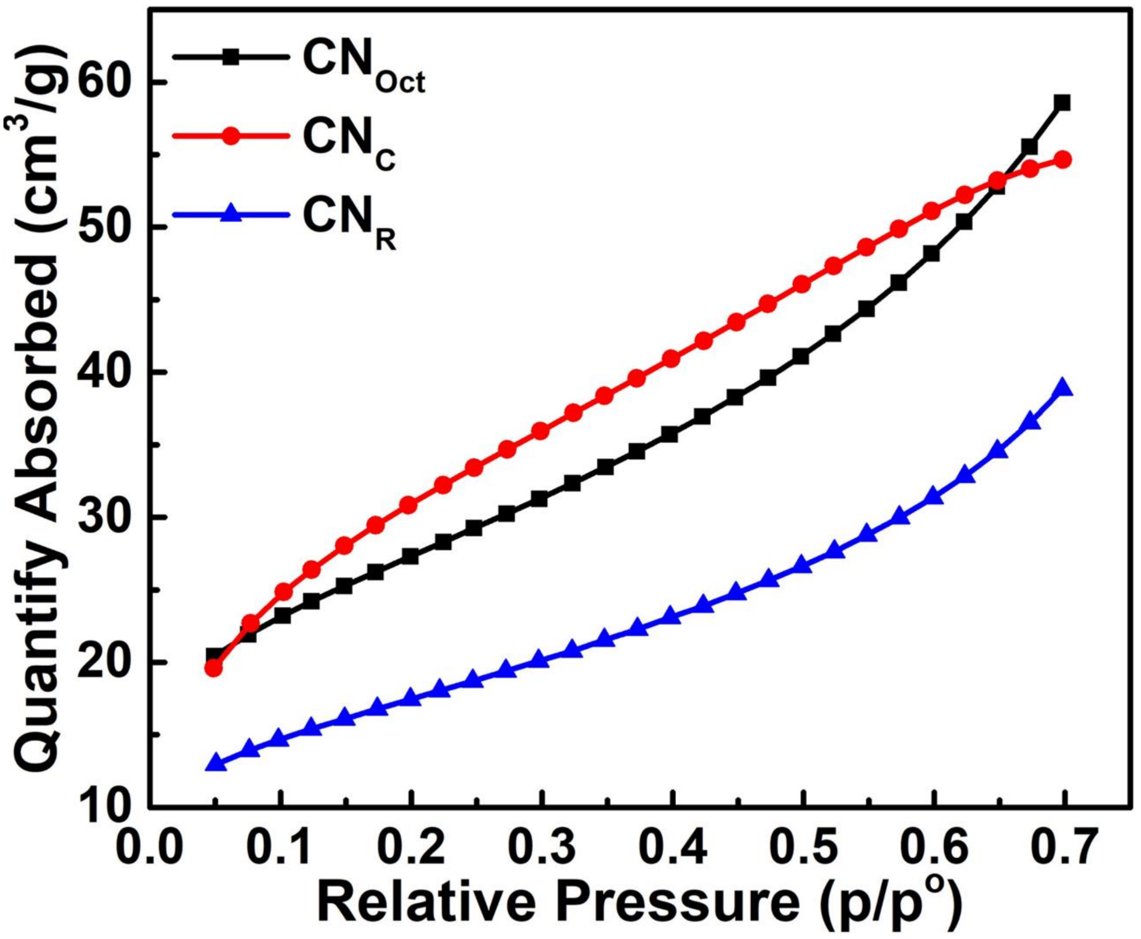

Figure S4. 27-point nitrogen absorption isotherm of the octahedral shaped CNP (CN_{Oct}), cubic shaped CNP (CN_C), rod shaped CNP (CN_R). The measured multipoint BET surface area are 98.65 ± 0.15, 116.97 ± 0.61, 63.28 ± 0.01 m^2/g for CN_{Oct}, CN_C, CN_R respectively.

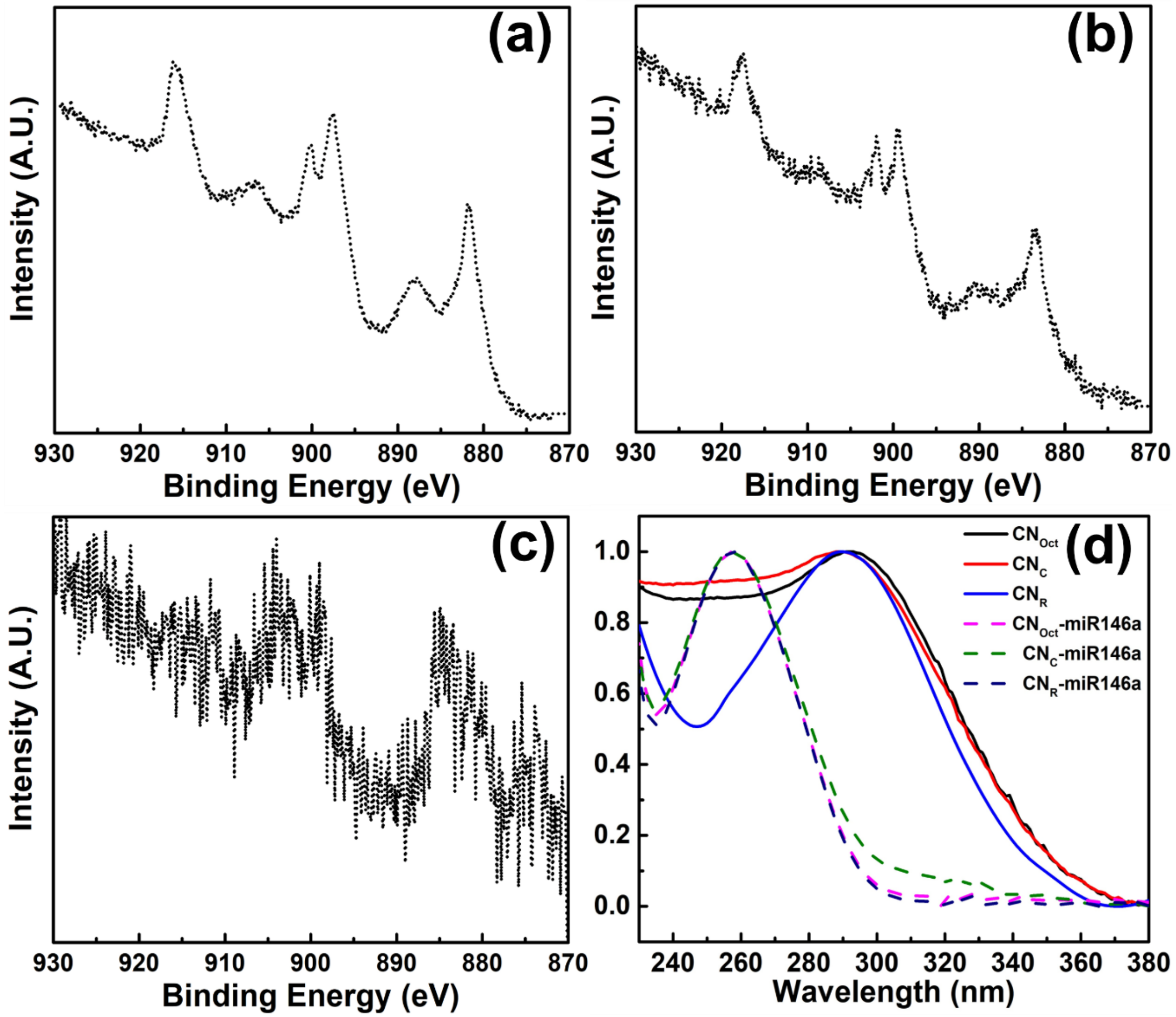

Figure S5. (a-c) Ce3d XPS spectrum of miRNA conjugated CNP. (a) Octahedral shaped CNP-miRNA conjugates. **(b)** Cubical shaped CNP-miRNA conjugates. **(c)** Rod shaped CNP-miRNA conjugates. No significant difference in Ce3d envelope were observed between bare CNP and miR146a conjugated CNP sample, indicating that Ce^{3+}/Ce^{4+} redox state ratio was not impacted by the miRNA146a conjugation process. **(d)** Nanoparticles synthesized in different shape, and it's conjugated with miRNA were subjected to UV-Vis analysis at room temperature to determine the conjugation of the miRNA molecules. Before conjugation, all bare nanoparticles showed absorption peak at 300 nm due to charge-transfer between O 2p and Ce 4f states. After miRNA conjugation, an absorption peak at 260 nm arises from the heterocyclic rings of the nucleotides associated with the conjugated miRNA146A.

Interpretation of the SOD/CAT% result from a different perspective

It is believed that the number of $V_{\cdot}^{O}$ sites is correlated with the number of Ce^{3+} site and $V_{\cdot}^{O}$ site is located adjacent to Ce^{3+} sites on the particle surfaces[4, 5]. Therefore, the density of $V_{\cdot}^{O}$ sites, the catalytic reactive sites for enzyme-mimetic reactions on CNPs surfaces, can be correlated to the fraction of Ce^{3+} (f_{Ce3+} =Ce^{3+} %) measured from XPS analysis. Further, the area occupied mostly by $V_{\cdot}^{O}$ site, (S_V), the sum of the area occupied mostly by all the $V_{\cdot}^{O}$ sites, can be correlated with the sum of the area occupied mostly by Ce^{3+} site (S_{Ce3+}) on the CNPs surface. Therefore S_{Ce3+} can be calculated by multiplying the CNPs surface area (S_{CN}), by f_{Ce3+}. If we assumed that the enzymatic mimic reaction only takes place at the area occupied mostly by $V_{\cdot}^{O}$ site, and this area can be defined as the "effective surface area" (S_{ESA})". Base on some basic relationship, and assuming that enzymatic activity of the CNPs is strongly depended on S_{ESA} , a positive correlation between S_{ESA} and SOD/CAT% can be derived below.

We know that,

$$S_{CN} = M_{CN} * S_{CN\text{-}BET}$$

(S_{CN}: CNPs surface area, M_{CN}: mass concentration of CNPs, g/mL, $S_{CN\text{-}BET}$=unit surface area of CNPs, m^2/g)

And,

$$S_{Ce3+} = S_{CN} * f_{Ce3+}$$

(S_{Ce3+}: area occupied mostly by Ce^{3+} site, S_{CN}: CNPs surface area, f_{Ce3+}: fraction of Ce^{3+} on CNPs surface)

Since,

$$S_V \propto S_{Ce3+},\ S_{ESA} \propto S_V, \text{ and SOD/CAT\%} \propto S_{ESA}$$

(S_V: area occupied mostly by $V_{\cdot}^{O}$ site, S_{Ce3+}: area occupied mostly by Ce^{3+} site, S_{ESA}: effective surface area, SOD/CAT%: enzymatic activity of CNPs)

Thus, S_{ESA} can be expressed and calculated from M_{CN}, and

$$\text{SOD/CAT\%} \propto S_{ESA} (\propto S_V \propto S_{Ce3+} = S_{CN} * f_{Ce3+} = M_{CN} * S_{CN\text{-}BET} * f_{Ce3+})$$

As an approximate value of S_{Ce3+} can be converted and calculated from each M_{CN} used in the SOD/CAT% assay, SOD/CAT% was plotted versus S_{Ce3+} in Figure. S6 to compare the enzymatic activities of the nanoparticle from shape to shape.

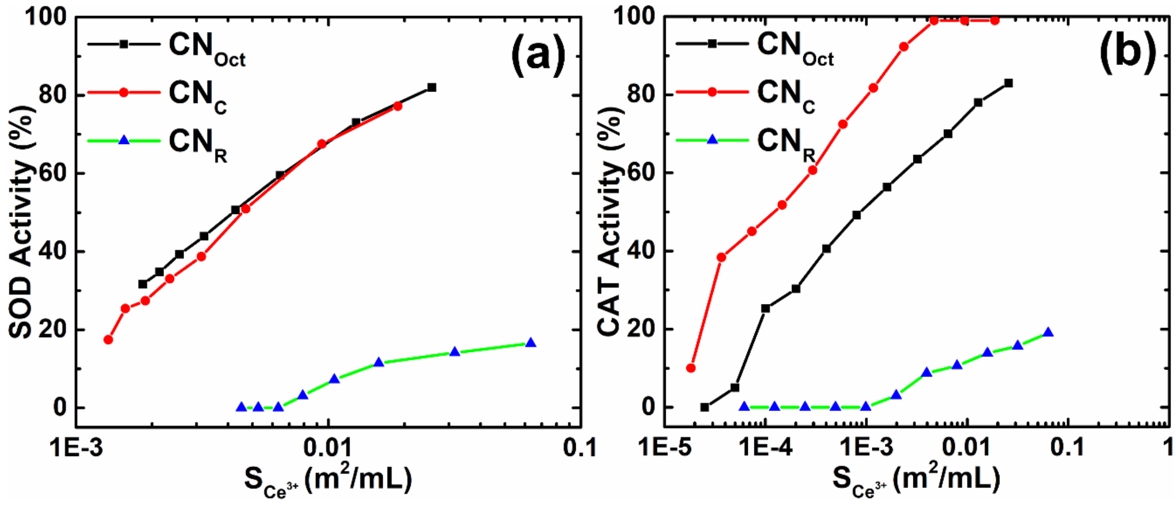

Figure S6. SOD and CAT% was plotted versus S_{Ce3+} in **(a)** and **(b)** respectively. Concept of S_{ESA} is introduced above to derive the associated relationship between SOD/CAT % and S_{Ce3+}.

In Figure S6a., a comparable SOD activity of CNPs was observed between CN_{Oct} and CN_{C}, CN_{R} displayed the lowest SOD activity, in Figure S6b., CN_{C} display the highest CAT activity, followed by CN_{Oct} and CN_{R}. As CN_{Oct}, CN_{C}, and CN_{R} exposed different lattice plane on the surface, (111), (100), (110) & (100), respectively, the normalized enzymatic activity analysis implies that (111) and (100) holds similar superoxide radical scavenging ability, and (100) is better than (111) in converting H_2O_2 into other species. Interestingly, the trend shown in here does not coincide much with many published studies.

DFT calculations[6-8] on reduced CeO_{2-x} surfaces, has indicated that per-oxo species ($O_2{}^{2-}$,H_2O_2) are more strongly bound to (111) than (100) & (110), which implies that (111) would be more favorable than (100) for H_2O_2 adsorption, a prerequisite step for the surface reaction. Therefore, CNPs which display (111) may have displayed a better CAT activity than which displays (100). Similarly, experimental studies [9-11], cited and discussed in the manuscript, has provided some comparable perspectives that (100) or (110) favors SOD reaction while (111) favors CAT reaction. These conclusions [9-11] have been drawn based on the SOD & CAT activity comparison between shapes of CNPs. Among these studies, it is not surprising that all the studies selected mass/molar concentration (M) as the controlled variable. Compared with other potential candidates, M is the easiest and of most convenience parameter to be controlled and varied in the SOD/CAT assay and many other experiments. Further, in these discussions, Ce^{3+}/Ce^{4+}% ratio was used as a critical factor to interpret the enzymatic activity difference between samples at fixed M but in various morphology. This is understandable as that, indeed, the Ce^{3+}/Ce^{4+}% ratio does play a key role in most cerium oxide material-related applications and can be assessed conveniently from XPS measurement. However, we cannot rule out the possibility that the conclusions drawn from these discussions inadvertently took the risk that, size or surface area could also contribute

a potentially non-negligible impact to their experimental data interpretation. On the other hand, even as in the above additional analysis, their analyses were made based on several assumptions, such as

- $V_{\cdot}^{O}$ are always formed on the surface.
- $V_{\cdot}^{O}$ are only created whenever and to wherever there is a need of charge compensation.
- $V_{\cdot}^{O}$ are always formed from the same type of mechanism.
- $V_{\cdot}^{O}$ are solely correlated by XPS measured Ce^{3+} %.

Based on above assumptions, we then build a model as a tool to interpret the experiment data. Inevitably, these assumptions were made to reduce the complexity of the built model by applying series of moderate extent of simplifications. However, in reality,

- $V_{\cdot}^{O}$ could be formed on the surface and the sub-surface [12].
- $V_{\cdot}^{O}$ could be created without the need of charge compensation [13].
- $V_{\cdot}^{O}$ could be created spontaneously and randomly from various type of defect formation mechanism during surface reconstruction.
- It is incomprehensive to solely correlate $V_{\cdot}^{O}$ with XPS measured Ce^{3+}%.

Therefore, it is the goal of all the researchers worked in this field to design a comprehensive model building strategy. Thereby, an objective and effective model can be created and used as a tool to evaluate the enzymatic activity of the nanoparticle.

Hence, in consideration of the excellent controllability and convenience of M among various candidates, we have also selected M as the controlled variable, in the first place, in designing the SOD & CAT% assay. Besides, as M was also used as a fixed variable in the miR146a conjugation protocol and biological studies, the SOD/CAT % comparison and discussion between samples was conducted and discussed at fixed M for the purpose of keeping the consistency. Therefore, fixed M was also considered as a fixed variable in the main manuscript discussion to interpret the experiment data and to associate the materials physicochemical properties with the result obtained from biological studies.

Reference

1. Scherrer, Paul. "Bestimmung Der Inneren Struktur Und Der Größe Von Kolloidteilchen Mittels Röntgenstrahlen." In *Kolloidchemie Ein Lehrbuch*, 387-409: Springer, 1912.
2. Langford, J Il, and AJC Wilson. "Scherrer after Sixty Years: A Survey and Some New Results in the Determination of Crystallite Size." *Journal of applied crystallography* 11, no. 2 (1978): 102-13.
3. Holzwarth, Uwe, and Neil Gibson. "The Scherrer Equation Versus The'debye-Scherrer Equation'." *Nature nanotechnology* 6, no. 9 (2011): 534-34.
4. Tyrsted, Christoffer, Kirsten Marie Ørnsbjerg Jensen, Espen Drath Bøjesen, Nina Lock, Mogens Christensen, Simon JL Billinge, and Bo Brummerstedt Iversen. "Understanding the Formation and Evolution of Ceria Nanoparticles under Hydrothermal Conditions." *Angewandte Chemie International Edition* 51, no. 36 (2012): 9030-33.
5. Tseng, Chun-Chieh, and Chih-Shan Li. "Inactivation of Viruses on Surfaces by Ultraviolet Germicidal Irradiation." *Journal of Occupational and Environmental Hygiene* 4, no. 6 (2007): 400-05.
6. Ji, Zhaoxia, Xiang Wang, Haiyuan Zhang, Sijie Lin, Huan Meng, Bingbing Sun, Saji George, Tian Xia, André E Nel, and Jeffrey I Zink. "Designed Synthesis of Ceo2 Nanorods and Nanowires for Studying Toxicological Effects of High Aspect Ratio Nanomaterials." *ACS nano* 6, no. 6 (2012): 5366-80.
7. Vinothkumar, G, Arun I Lalitha, and K Suresh Babu. "Cerium Phosphate–Cerium Oxide Heterogeneous Composite Nanozymes with Enhanced Peroxidase-Like Biomimetic Activity for Glucose and Hydrogen Peroxide Sensing." *Inorganic chemistry* 58, no. 1 (2018): 349-58.
8. Shoko, E, MF Smith, and Ross H McKenzie. "Charge Distribution near Bulk Oxygen Vacancies in Cerium Oxides." *Journal of Physics: Condensed Matter* 22, no. 22 (2010): 223201.
9. Ganduglia-Pirovano, M Veronica, Juarez LF Da Silva, and Joachim Sauer. "Density-Functional Calculations of the Structure of near-Surface Oxygen Vacancies and Electron Localization on Ceo 2 (111)." *Physical review letters* 102, no. 2 (2009): 026101.
10. Mullins, David R. "The Surface Chemistry of Cerium Oxide." *Surface Science Reports* 70, no. 1 (2015): 42-85.
11. Zhao, Yun, Bo-Tao Teng, Xiao-Dong Wen, Yue Zhao, Qiao-Ping Chen, Lei-Hong Zhao, and Meng-Fei Luo. "Superoxide and Peroxide Species on Ceo2 (111), and Their Oxidation Roles." *The Journal of Physical Chemistry C* 116, no. 30 (2012): 15986-91.
12. Nolan, Michael. "Healing of Oxygen Vacancies on Reduced Surfaces of Gold-Doped Ceria." *The Journal of Chemical Physics* 130, no. 14 (2009): 144702.
13. Li, Yuanyuan, Xiao He, Jun-Jie Yin, Yuhui Ma, Peng Zhang, Jingyuan Li, Yayun Ding, Jing Zhang, Yuliang Zhao, and Zhifang Chai. "Acquired Superoxide-Scavenging Ability of Ceria Nanoparticles." *Angewandte Chemie* 127, no. 6 (2015): 1852-55.
14. Naganuma, Tamaki. "Shape Design of Cerium Oxide Nanoparticles for Enhancement of Enzyme Mimetic Activity in Therapeutic Applications." *Nano Research* 10, no. 1 (2017): 199-217.
15. Yang, Yushi, Zhou Mao, Wenjie Huang, Lihua Liu, Junli Li, Jialiang Li, and Qingzhi Wu. "Redox Enzyme-Mimicking Activities of Ceo2 Nanostructures: Intrinsic Influence of Exposed Facets." *Scientific reports* 6, no. 1 (2016): 1-7.
16. Namai, Yoshimichi, Ken-Ichi Fukui, and Yasuhiro Iwasawa. "Atom-Resolved Noncontact Atomic Force Microscopic and Scanning Tunneling Microscopic Observations of the Structure and Dynamic Behavior of Ceo2 (1 1 1) Surfaces." *Catalysis today* 85, no. 2-4 (2003): 79-91.
17. Choi, YongMan, Harry Abernathy, Hsin-Tsung Chen, Ming-Chang Lin, and Meilin Liu. "Characterization of O2–Ceo2 Interactions Using in Situ Raman Spectroscopy and First-Principle Calculations." *ChemPhysChem* 7, no. 9 (2006): 1957-63.
